# Development of a targeted amplicon sequencing method for genotyping *Cyclospora cayetanensis* from fresh produce and clinical samples with enhanced genomic resolution and sensitivity

**DOI:** 10.3389/fmicb.2023.1212863

**Published:** 2023-06-16

**Authors:** Susan R. Leonard, Mark K. Mammel, Baback Gharizadeh, Sonia Almeria, Zhihai Ma, David J. Lipman, Mary E. Torrence, Chunlin Wang, Steven M. Musser

**Affiliations:** ^1^Office of Applied Research and Safety Assessment, Center for Food Safety and Applied Nutrition, U.S. Food and Drug Administration, Laurel, MD, United States; ^2^Chapter Diagnostics, Menlo Park, CA, United States; ^3^Center for Food Safety and Applied Nutrition, U.S. Food and Drug Administration, College Park, MD, United States

**Keywords:** cyclosporiasis, targeted amplicon sequencing, epidemiology, foodborne parasite, bait-capture, genetic clustering

## Abstract

Outbreaks of cyclosporiasis, an enteric illness caused by the parasite *Cyclospora cayetanensis*, have been associated with consumption of various types of fresh produce. Although a method is in use for genotyping *C. cayetanensis* from clinical specimens, the very low abundance of *C. cayetanensis* in food and environmental samples presents a greater challenge. To complement epidemiological investigations, a molecular surveillance tool is needed for use in genetic linkage of food vehicles to cyclosporiasis illnesses, estimation of the scope of outbreaks or clusters of illness, and determination of geographical areas involved. We developed a targeted amplicon sequencing (TAS) assay that incorporates a further enrichment step to gain the requisite sensitivity for genotyping *C. cayetanensis* contaminating fresh produce samples. The TAS assay targets 52 loci, 49 of which are located in the nuclear genome, and encompasses 396 currently known SNP sites. The performance of the TAS assay was evaluated using lettuce, basil, cilantro, salad mix, and blackberries inoculated with *C. cayetanensis* oocysts. A minimum of 24 markers were haplotyped even at low contamination levels of 10 oocysts in 25 g leafy greens. The artificially contaminated fresh produce samples were included in a genetic distance analysis based on haplotype presence/absence with publicly available *C. cayetanensis* whole genome sequence assemblies. Oocysts from two different sources were used for inoculation, and samples receiving the same oocyst preparation clustered together, but separately from the other group, demonstrating the utility of the assay for genetically linking samples. Clinical fecal samples with low parasite loads were also successfully genotyped. This work represents a significant advance in the ability to genotype *C. cayetanensis* contaminating fresh produce along with greatly expanding the genomic diversity included for genetic clustering of clinical specimens.

## Introduction

*Cyclospora cayetanensis* is a coccidian parasite that causes cyclosporiasis, an enteric illness, in humans (Almeria et al., 2019; Mathison and Pritt, 2021). In recent years, reported cyclosporiasis outbreaks have been increasing,^1^ including 2,299 laboratory-confirmed illnesses in multiple outbreaks in the United States in 2018 (Casillas et al., 2018) and 2,408 illnesses in 2019 (Barratt et al., 2021). Outbreaks of cyclosporiasis have been linked to consumption of fresh produce, predominately leafy greens, berries, and herbs (Almeria et al., 2019; Hadjilouka and Tsaltas, 2020). Although many cases of cyclosporiasis in the United States have been traced back to imported fresh produce, *C. cayetanensis* has been detected in samples of domestically grown romaine lettuce and cilantro (Gottlieb, 2018). A method for genotyping *C. cayetanensis* contaminating fresh produce or environmental samples with sufficient genetic resolution would aid in identifying the food vehicle or agricultural region associated with a cyclosporiasis outbreak without relying solely on epidemiological data. Whole genome sequencing (WGS) has been successfully employed for foodborne bacterial pathogens as a molecular surveillance tool by public health agencies in combination with epidemiological data to link food items and agricultural areas or processing facilities with clinical illnesses (Allard et al., 2016; Stevens et al., 2022). However, WGS is not a practical option for obtaining genetic cluster information for *C. cayetanensis* as the parasite cannot be propagated in the laboratory and the low levels of contamination in food and water preclude isolation (Almeria et al., 2019). Molecular surveillance methods for *C. cayetanensis* must, therefore, be developed for use with culture independent metagenomic samples.

While sensitive and specific detection of *C. cayetanensis* in produce is possible (Balan et al., 2023), tools for genotyping *C. cayetanensis* contaminating food and environmental samples have been lacking. In part, this is due to the typically lower levels of *C. cayetanensis* contamination in food and surface water samples compared to fecal samples. Even in clinical samples, development of genotyping tools has been challenging and slow. Early attempts to perform multi-locus sequencing typing (MLST) based on five microsatellite markers using either nested PCR or one-step PCR resulted in relatively low rates of amplification, difficult to interpret sequence results due to short repeat units, and poor discriminatory power (Guo et al., 2016; Hofstetter et al., 2019). More recently, to supplement epidemiological information when investigating outbreaks, the U.S. Centers for Disease Control and Prevention (CDC) has developed a different MLST scheme for genetic clustering of *C. cayetanensis* in clinical fecal samples (Nascimento et al., 2020). This MLST approach combines eight previously described targets, including two discriminatory locations in the *C. cayetanensis* mitochondrial genome and six in the nuclear genome (Barratt et al., 2019; Nascimento et al., 2019; Houghton et al., 2020). It has been used for genotyping clinical samples containing *C. cayetanensis* associated with major outbreaks in the United States (Barratt et al., 2021), and recently was also applied to fecal samples from Canadian cyclosporiasis illnesses (Yanta et al., 2022). While the eight markers included in the MLST scheme used by the CDC have proven to be a useful step forward for genotyping, it has become apparent that including additional markers to increase coverage of phylogenetically informative single nucleotide polymorphisms (SNPs) is necessary to more fully and accurately resolve linkages between samples (Barratt et al., 2022; Yanta et al., 2022). The MLST genotyping method involves performing conventional PCR for each marker individually prior to combining amplicons for sequence library preparation, therefore increasing the number of markers necessitates an increased total volume of sample DNA. This would be problematic for genotyping *C. cayetanensis* in contaminated food samples due to the limited quantity of DNA obtained when recovering the microbiome from the sample combined with the low relative abundance of *C. cayetanensis* in the microbiome.

Targeted amplicon sequencing (TAS) greatly enhances sensitivity for obtaining sequence for target genes compared to shotgun metagenomic sequencing and has been used in a variety of applications where specific loci of interest are amplified in a highly multiplexed reaction. For example, it has been utilized for culture independent detection and partial characterization of viral pathogens in human clinical samples, animal vectors, and wastewater (Kuchinski et al., 2022; Ma et al., 2022; Silva et al., 2022), as well as for geographical segregation based on SNP panels and for drug resistance surveillance of *Plasmodium vivax* parasites in human blood samples (Kattenberg et al., 2022). For *C. cayetanensis*, greater than 98% of the mitochondrial genome sequence was obtained from fresh produce samples inoculated with low levels of oocysts using a TAS assay designed to target the entire mitochondrial genome (Cinar et al., 2022). However, phylogenetic resolution is very limited when including only mitochondrial sequence variation among samples. Including both the 40 *C. cayetanensis’* WGS assemblies and 35 additional mitochondrial genomes at NCBI, the mitochondrial genome contains a total of only 17 SNP positions. An assay targeting markers in the nuclear genome is necessary but presents a greater challenge as it is estimated that the ratio of mitochondrial to nuclear genomes is at least 67 to 1 (Cinar et al., 2022) or greater (Tang et al., 2015). Low levels of target template DNA can result in a significant quantity of off-target amplification and/or primer dimers. Despite the challenges, given the limited DNA volume obtained from food samples and the need for increased phylogenetic cluster resolution for linkage of food vehicles with clinical illnesses, TAS offers an attractive option for genotyping *C. cayetanensis* if additional markers in the nuclear genome can be included.

An alternative to TAS for enriching metagenomic sequencing libraries for genomic loci of interest is the use of a hybridization capture method. In this technique, often referred to as bait-capture, sequence-specific single-stranded oligonucleotides (baits) are combined with DNA fragments. The baits hybridize with DNA fragments containing the targeted loci, are captured, and non-targeted DNA fragments are depleted in wash steps (Fitzpatrick et al., 2021; Singh, 2022). Bait-capture assays have been employed for a variety of applications, for example, genotyping the human pathogen *Leptosira interrogans* from clinical samples using baits spanning the entire core genome (Grillova et al., 2023), and for obtaining *Mycobacterium tuberculosis* genomes directly from clinical specimens (Brown et al., 2015). To our knowledge, the use of a hybridization capture method for genotyping *C. cayetanensis* has not been reported.

In this study, we develop a novel TAS assay for genotyping *C. cayetanensis* that includes amplification of all markers in a single multiplexed PCR. The design contains a panel of markers that greatly increase the number of informative SNPs in the nuclear genome in comparison to currently used genotyping assays and, with an added bait-capture step in the workflow, allows for genotyping at low levels of *C. cayetanensis* oocyst contamination. Using commercial Ready-to-Eat (RTE) chopped romaine lettuce samples inoculated with various levels of *C. cayetanensis* oocysts, we determine the sensitivity of the genotyping assay, and include other relevant fresh produce items to investigate comprehensiveness. The ability to amplify many targets for genotyping from suboptimal clinical fecal samples is also demonstrated, and we evaluate the utility of the tool as a molecular method to complement epidemiological investigations.

## Materials and methods

### Fresh produce sample preparation

Commercial fresh bagged chopped romaine lettuce was purchased from a local grocery store and used before the expiration date. Romaine lettuce samples (25 g each) were inoculated with a preparation of purified *C. cayetanensis* oocysts from a patient in Guatemala. Purified oocysts that had been stored in 0.25% potassium dichromate were washed three times in 0.85% sodium chloride and concentrated by centrifugation. Six replicates of the concentrated oocyst preparation were enumerated using a hemocytometer. Based on the enumeration results, dilutions were prepared in 0.85% sodium chloride to obtain known concentrations of oocysts for inoculation. Individual romaine lettuce samples were inoculated with either 10, 20, 50, 100, or 200 oocysts. The oocysts were randomly spread in approximately 10–20 droplets across the sample surface with a micropipette. Inoculation, washing the romaine lettuce samples to obtain the microbiome, and DNA extraction followed the procedures previously described for salad mix samples (Almeria and Shipley, 2021). Two technical replicates were performed with the TAS assay from each of the five DNA samples. A variety of inoculated fresh produce items including salad mix (romaine and iceberg lettuces, carrots, and red cabbage), cilantro, and basil samples were also prepared using the same procedure. In addition, a blackberry sample (50 g) was inoculated with 10 oocysts and processed as previously described (Assurian et al., 2020). Oocysts from a patient diagnosed with cyclosporiasis in Indonesia were used for inoculating the salad mix, basil, and blackberries. The purified oocysts from clinical specimens from Guatemala and Indonesia were generously supplied by the CDC, and use of these oocysts was approved by the Institutional Review Board of the FDA (Protocol 15-039F and RIHSC-ID#10-095F).

### Clinical sample DNA isolation

Clinical fecal samples from humans diagnosed with cyclosporiasis were kindly supplied by the Texas Department of Health (HHSF223201810028I-75F40119F19007). DNA was extracted from the samples using the FASTDNA SPIN Kit for soil along with bead-beating using the FastPrep-24 instrument (MP Biomedicals, Santa Ana, CA) according to the manufacturer’s protocol with modifications as previously described (Balan et al., 2023). The fecal samples had been stored at −20°C prior to use.

### Metagenomic sample DNA for primer development

Five fresh produce and four surface water samples were inoculated with *C. cayetanensis* oocysts at low and high levels for use in testing primers during TAS assay development. DNA from inoculated fresh produce samples (10–200 oocysts) was prepared as stated in Fresh Produce Sample Preparation above and from inoculated water samples (200–20,000 oocysts) as previously described (Durigan et al., 2020), with the modification that 50 L of water was filtered for each sample. Aliquots of the DNA were shipped to Chapter Diagnostics for use in laboratory testing during the primer design process.

### Candidate marker identification and primer design

At the time of the TAS design, 40 WGS assemblies of *C. cayetanensis* were available at NCBI and were used as a database for discovery of informative markers. Two approaches were used to identify potential markers. First, *C. cayetanensis* isolate NF1_C8 Nepal (accession PVNT01000000) was used as a reference genome from which the nucleotide sequences of 5,793 annotated proteins were obtained. The nucleotide sequences were then used in BLAST queries against the database of WGS assemblies. Defining core genes as those present only one time in an assembly and contained in at least 35 of the 40 assemblies, 3,460 core genes were identified. Custom Python scripts were used to scan the BLAST matched sequences for each core gene to discover SNP positions. Potential markers for the assay were evaluated by identifying 100 bp regions containing SNPs flanked by conserved regions, and the information content of the SNP region was determined by calculating the Shannon entropy of the frequency of the groups of SNP patterns seen in the WGS sequences. The 250 potentially informative markers that were identified were pruned both by removal of sequences matching by BLAST to *Eimeria* species and by entropy scores, for a final list of 71 markers.

In a second approach for potential marker identification, contigs for *C. cayetanensis* isolate NF1 (accession MSEL00000000) were concatenated as a reference genome from which fragments between 280 and 350 bases were obtained. Fragments were used as BLAST queries against the 40 WGS assemblies of *C. cayetanensis*, and fragments with BLAST hits in all 40 WGS assemblies were saved. The entropy was calculated for each fragment and fragments with entropy values >1.0 were considered for inclusion. Thirty fragments evenly located along the artificial genome were then picked as templates for primer design. The list of 71 markers from the FDA was further pruned to 24 by keeping only those present in ≥38 WGS assemblies and with entropy values >1.0. Markers for inclusion in the first design attempt were chosen from the two sets of candidate markers. In addition to the combined set of 54 markers, 11 markers were added to cover the majority of the virtual amplicons included in the eight marker MLST assay in use by the CDC (Nascimento et al., 2020). Multiple sequence alignments were constructed for the eight loci using the 40 WGS assemblies. Two markers each were designed within the HC378, HC360i2, and MSR loci, and one marker each was designed to cover the remaining five loci (CDS1, CDS2, CDS3, CDS4, and MT-junction). Primers were designed for the set of chosen markers and then tested in the TAS assay in the laboratory using DNA extracted from fresh produce and surface water samples inoculated with *C. cayetanensis* oocysts. Primers were re-designed or dropped as needed to optimize amplification of *C. cayetanensis* markers and reduce off-target amplification and primer dimers.

### Real-time PCR for *Cyclospora cayetanensis*

Amplification and quantitation of *C. cayetanensis* specific DNA was performed as previously described using Real-time PCR in a duplex reaction, targeting both the specific *C. cayetanensis cox3* gene located in the mitochondrial genome (Mit1C target) and an exogenous internal amplification control (Balan et al., 2023). Real-time PCR was performed in an Applied Biosystems 7500 Fast Real time PCR System (ThermoFisher Scientific, Waltham, MA). A commercially prepared synthetic gBlocks DNA fragment (Integrated DNA Technologies, Coralville, CA) was used as a positive control for amplification of *C. cayetanensis,* and a no template control reaction was included as a negative control. Experimental samples were considered positive when one or more of three technical replicates produced a positive result with a cycle threshold (C_T_) ≤ 38.0 for the Mit1C target (Balan et al., 2023).

### Targeted amplicon sequencing

TAS was performed using the ChapterDX *Cyclospora* Target Enrichment NGS Assay kit (Chapter Diagnostics, Menlo Park, CA) according to the manufacturer’s protocol. Briefly, for each sample, 5 uL DNA was used in a single multiplex PCR containing target-specific primers for all amplicons. After purification using SPRIselect beads (Beckman Coulter, Brea, CA), the amplified product for each sample was subjected to a bait-capture step utilizing biotin labeled probes captured with Dynabeads MyOne Streptavidin T1 (Invitrogen, Waltham, MA). A 35 min hybridization of amplicons and baits was used in this work. Amplification and addition of barcodes and adapters for use with Illumina sequencing platforms (Illumina, San Diego, CA) was performed on the captured amplicons in a second PCR, generating the sequencing libraries. Only half of the bait-capture enriched targeted amplicons (5 μL) are used as template in this indexing PCR step and the second half was stored at −20°C as a backup. A Bio-Rad S1000 Thermal Cycler (Bio-Rad, Hercules, CA) was used for both PCR steps. Following a SPRIselect bead purification step, the libraries were quantitated using the Qubit High Sensitivity Assay (Qubit, London, United Kingdom) and inspected for quality using the Agilent TapeStation 4150 (Agilent, Santa Clara, CA). Libraries for six samples were pooled in an Illumina MiSeq run yielding paired-end 249 bp reads. Negative controls including sterile water and uninoculated fresh produce samples yielded libraries too dilute for sequencing and without expected library fragment sizes.

### Sequence analysis

Fastq files were quality controlled with FastQC^2^ and then to ensure quality across the length of the marker prior to haplotyping, the paired-end reads were merged using PEAR: a fast and accurate Illumina Paired-End reAd merger (Zhang et al., 2014). To determine haplotypes from the merged reads, the reads were BLASTed against a database of haplotype sequences. To be considered a haplotype match, a read was required to cover all SNPs within the amplicon with no mismatches. New haplotypes of sufficient frequency were detected and added to the database. The percentage of each haplotype assigned to a given amplicon was determined by comparing the number of reads matching the haplotype to the total number of reads for the amplicon. Haplotypes were called present if there were at least 10 matching reads and the matching reads constituted at least 10% of the total reads for the amplicon. The Eukaryotyping program (R scripts) from the CDC (Barratt et al., 2019) was used to generate a pairwise distance matrix of the samples from the haplotype presence/absence results. The program uses two methods, Bayesian and Heuristic, to build distance matrices. In this work, only the Heuristic matrix was used for clustering. Hierarchical clustering was performed in R using Agnes from the ‘cluster’ package version 2.1.4 using Manhattan distances and the Ward clustering method.^3^

To determine the number of reads matching genera in the family *Eimeriidae*, low quality reads were trimmed using Trimmomatic (Bolger et al., 2014) with a threshold of Q > 30. Trimmed reads were matched using MegaBLAST (Altschul et al., 1990) to a custom *Eimeriidae* database composed of 91 mitochondrial genomes and 41 WGS assemblies. To remove nonspecific reads, the reads matching *Eimeriidae* were queried against the NCBI Nucleotide collection (nt) using MegaBLAST and reads with a higher BLAST score to other sequences compared to *Eimeriidae* were removed from the count.

To construct the SNP-based phylogenetic trees from the 40 *C. cayetanensis* assemblies at NCBI for the purpose of comparing clustering differences using the whole genome and the TAS assay, alleles of each gene or amplicon were retrieved from the NCBI assemblies by BLAST match to loci in the NF1_C8 Nepal assembly and aligned with ClustalW version 2.1 using the Slow/Accurate option and default parameters (Larkin et al., 2007). Some genes have two alleles in the WGS assemblies and for the core gene analysis, only genes occurring once in the chromosomal genome assembly were used. This eliminated either possible paralogs or possible duplication in the assembly due to heterozygosity in that gene for the sequenced isolate. For the loci included in the TAS assay, if there was more than one allele in the assembly for a locus, the allele sequence with the highest BLAST score was used to construct the phylogeny. Alignments were scanned with a custom Python program to determine SNP positions and produce a FASTA file of concatenated SNPs for each genome. Phylogenetic trees were created with MEGA version 10.0.5 (Kumar et al., 2018). The evolutionary history was inferred using the neighbor-joining method (Saitou and Nei, 1987), and evolutionary distances were computed using the p-distance method (Nei and Kumar, 2000) with pairwise deletion of gaps/missing data and otherwise default parameters. For visual comparison, the two phylogenetic trees were imported into R in Newick format and a tanglegram was generated using the package dendextend (Galili, 2015). Some branches were rotated using the click-rotate function to minimize crossing edges.

## Results

### Genotyping markers included in the assay

An initial set of candidate markers for genotyping were chosen and primers were designed for a multiplex PCR targeting all chosen loci. The primer panel was tested on DNA from metagenomic samples of fresh produce and surface water inoculated with varying levels of *C. cayetanensis* oocysts. Problematic primers resulting in off-target amplification or excessive primer dimers were either re-designed or removed from the panel. After two iterations of design and testing, 52 candidate markers were chosen for inclusion in the TAS panel (Table 1). Amplicon sizes range from 220 to 376 bp and together cover a total of 14,264 bp. Markers CG, CK, and CH are located in the mitochondrial genome and all other markers are contained in the nuclear genome. Instead of genomic variation based on SNP positions, marker AU includes two indels and marker CH contains a variable repeat region. During the design of the TAS assay, the number of SNP sites within markers as well as the number of haplotypes represented for each marker were assessed using the 40 *C. cayetanensis* WGS assemblies available at NCBI (Table 1). While the assay includes 356 currently known SNP positions when considering only the 40 WGS assemblies at NCBI, additional SNP locations within the markers have been identified as clinical samples have been genotyped using the kit, resulting in a current total of 396 SNP positions. As one measure of informativeness, the Shannon entropy for each marker was computed using the distribution of haplotypes among assemblies and samples and, in some cases, increased, while in others decreased, as additional sample results were included with the assemblies at NCBI.

**Table 1:** Description of markers included in the targeted amplicon sequencing assay.

Marker	Length (bp)	SNPs (NCBI)	SNPs (NCBI plus)^1^	Haplotypes (NCBI)	Haplotypes (NCBI plus)^1^	Entropy (NCBI)	Entropy (NCBI plus)^1^
AA	325	4	4	4	4	1.496	1.363
AC	376	21	21	8	9	2.229	2.285
AD	264	15	16	7	11	2.368	2.376
AE	339	21	21	10	10	2.932	2.858
AF	253	14	19	8	10	2.416	2.458
AG	267	6	7	5	8	2.206	2.102
AH	288	3	3	4	4	1.679	1.713
AJ	361	5	7	3	5	1.485	1.676
AK	355	7	8	4	4	1.550	1.616
AL	271	8	8	4	4	1.695	2.012
AM	375	8	9	4	5	1.483	1.671
AO	370	9	13	6	11	2.321	2.225
AP	315	3	4	3	5	1.489	1.463
AQ	238	3	3	3	3	1.568	1.597
AR	315	6	6	3	3	1.451	1.662
AS	293	3	4	3	5	1.543	1.758
AU^2^	262	0	0	3	3	1.500	1.576
AV	325	6	7	4	7	1.629	2.444
AW	243	3	3	3	3	1.552	1.526
AX	236	4	4	3	3	1.493	2.695
AY	229	2	3	3	6	1.493	1.698
AZ	267	6	6	5	5	2.006	2.137
FA	299	13	16	10	10	2.820	2.980
FB	224	6	6	9	10	2.900	2.839
FC	250	7	7	8	10	2.618	2.419
FD	312	13	15	8	12	2.726	2.879
FE	225	4	4	5	5	1.507	1.773
FF	235	3	3	4	7	1.486	1.976
FG	256	10	10	6	6	1.713	2.040
FH	256	7	9	5	10	1.620	2.822
FI	292	13	15	6	9	1.985	2.508
FL	221	3	4	4	5	1.676	1.872
FM	247	7	7	5	6	1.937	1.590
FN	236	4	4	5	8	1.893	2.281
FP	275	5	6	5	7	1.791	1.683
FQ	302	7	8	4	6	1.722	1.551
FR	255	5	5	6	6	1.999	1.649
FT	252	4	4	4	4	1.679	1.430
FU	248	8	8	5	6	1.789	2.000
FV	228	3	4	3	4	1.526	1.862
FW	222	4	4	4	4	1.572	1.776
CA	328	12	12	3	3	0.707	0.687
CB	295	2	4	2	4	0.996	1.097
CC	279	2	2	2	2	0.984	0.956
CD	334	4	5	2	3	0.769	0.987
CE	354	17	18	6	6	1.731	1.824
*CF*	220	6	8	9	10	2.474	2.743
CG	270	3	4	4	5	1.706	2.198
CI	266	12	14	6	9	1.683	1.764
CJ	250	12	12	12	15	2.961	3.506
CK	266	2	2	3	3	1.222	1.542
CH^3^				6	7	2.022	2.157

1 Includes WGS assemblies at NCBI and all clinical samples or purified oocysts analyzed with the assay.

2 AU contains two indel positions.

3 CH contains a variable repeat region.

Eleven markers in the TAS assay target areas of the *C. cayetanensis* genome covered by the eight loci MLST scheme currently in use by the CDC (Nascimento et al., 2020). Detailed inspection of these loci included in the two assays was performed to determine the extent of overlap and SNP sites included (number of SNP sites is based on the 40 WGS assemblies at NCBI) (Supplementary Table S1). The sequence regions covered by the markers in the genotyping assays are not completely identical. The loci for markers CDS1, CDS2, CDS3, and CDS4 are entirely overlapped by markers in the TAS assay, while there are regions within markers HC378, HC360i2, and MSR that are not included in the TAS assay. Some TAS assay markers extend into the genomic regions flanking the eight-loci MLST markers and contain additional SNP positions. Altogether, the TAS assay includes 54 of the 58 SNP sites contained in the MLST assay. In addition, there are 18 SNP sites within the 11 TAS markers that are not represented in the MLST assay (Supplementary Table S1). It is noteworthy that four of the markers, CA, CB, CC, and CD display the lowest entropy values of the 52 markers in the TAS assay (Table 1) and would not have passed the criteria for inclusion (entropy >1.0) had they not been included as part of the MLST eight-loci panel for consistency and comparative purposes between the two genotyping panels.

### Comparison of chromosomal core gene and TAS marker phylogenies

**Figure 1:**
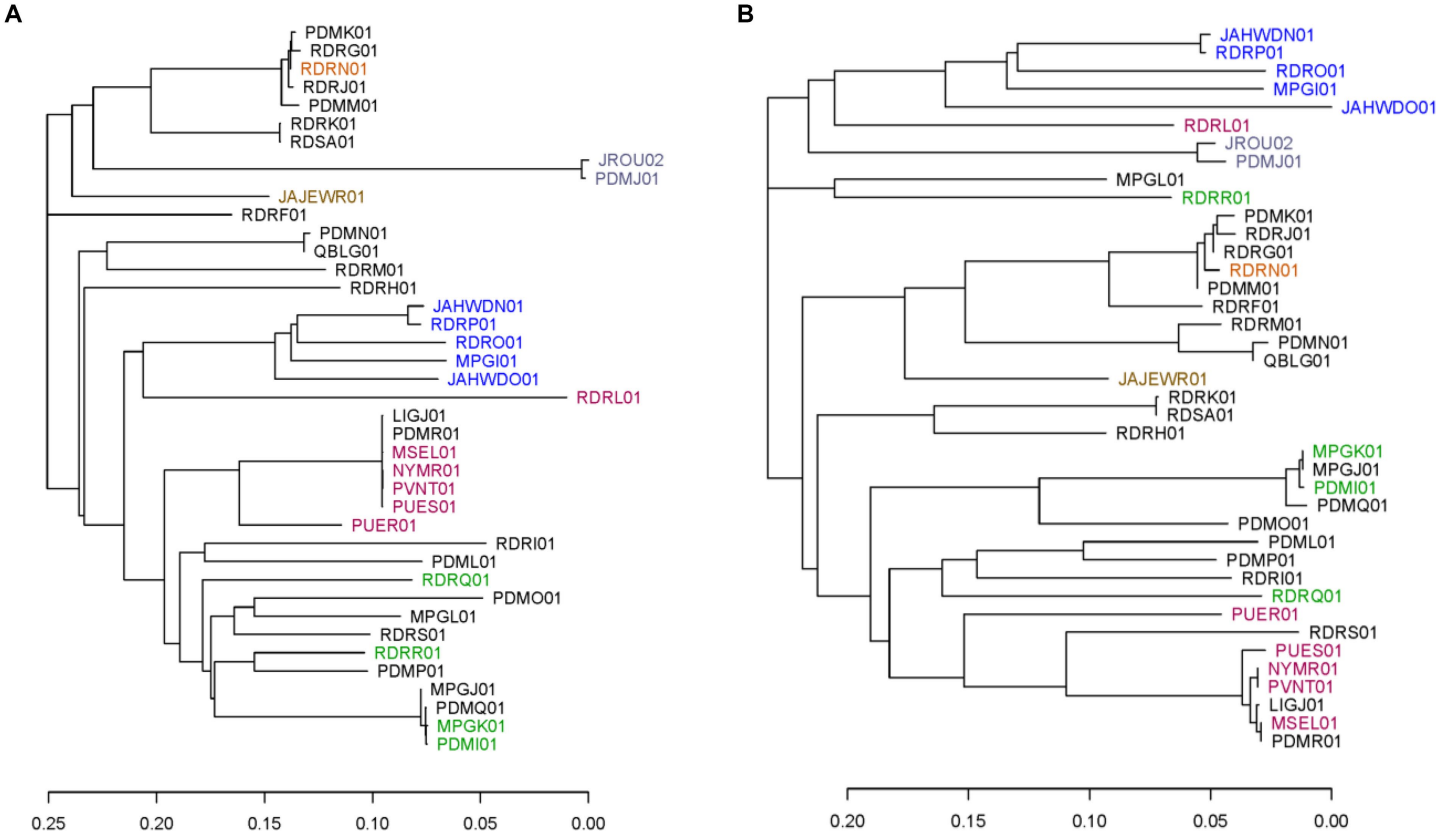
Phylogenetic trees including the 40 publicly available *C. cayetanensis* whole genome sequence assemblies. Phylogenies were created using the neighbor-joining method based on SNPs included in **(A)** all core chromosomal genes and **(B)** markers in the TAS assay described herein. Accession numbers are used to label the assemblies and are color coded based on the source country: black, United States; orange, Mexico; lavender, China; brown, Canada; blue, Indonesia; red, Nepal; green, Guatemala. The scale portrays the number of base differences per site.

To evaluate the marker panel further, phylogenetic trees were created using the neighbor-joining method based on SNPs within the 40 available *C. cayetanensis* WGS assemblies for the core chromosomal genes and the TAS assay markers described herein. Markers AU and CH in the TAS assay were excluded from this examination since the sequence variation is not SNP-based. Analysis revealed 3,460 core chromosomal genes that include 39,071 SNP sites in the WGS assemblies and 355 SNPs in the 50 markers included in the TAS assay. Whereas the TAS assay included five SNPs in the mitochondrial genome in this analysis, the core gene phylogeny included only chromosomal loci. In addition, the core gene analysis was based on SNPs within genes, while SNPs in markers within intergenic regions were also included for the TAS genotyping method. The TAS assay encompasses markers within 26 chromosomal core genes. To a great extent, at a fairly low resolution, the phylogenetic tree based on the SNP sites in the TAS assay recapitulates the tree based on SNPs in the 3,460 core genes (Figures 1A,B). Comparison of the two trees demonstrates that although at a finer resolution the branching is different for many isolates, the major clusters are preserved (Supplementary Figure S1). In particular, similar geographic segregations are attained. For example, the Indonesian isolates cluster from one branch in the tree. Isolates that are short distances within clusters in the core gene tree are also clustered tightly in the TAS assay tree. There are, however, individual isolates that cluster very disparately between the trees, namely, a Guatemalan isolate RDRR01 and United States:RI isolate MPGL01.

### Addition of bait-capture step to the targeted amplicon sequencing assay

**Figure 2:**
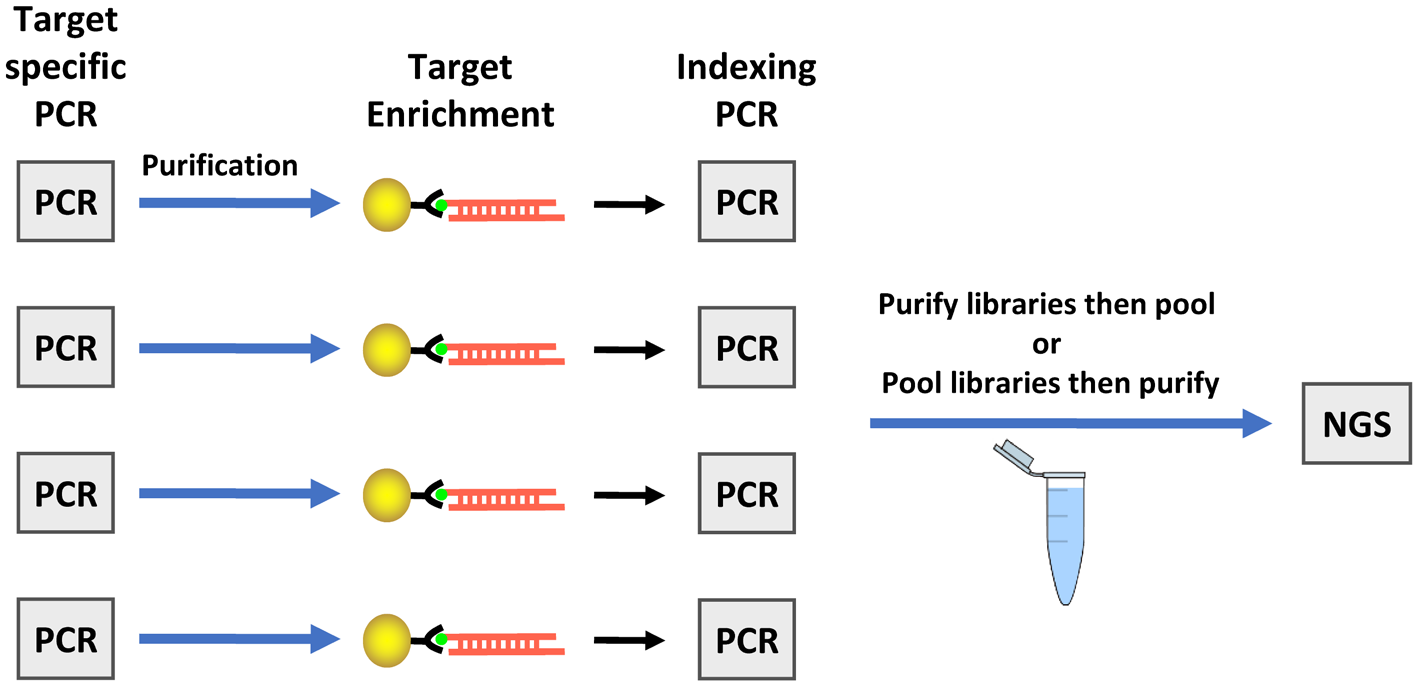
Workflow for the targeted amplicon sequencing (TAS) with additional bait-capture enrichment method. The illustration depicts the workflow for four samples as an example, however, simultaneous preparation of enriched TAS libraries is not limited to four samples. The target specific PCR includes primers for all markers in one tube. Next generation sequencing (NGS) is performed using an Illumina platform sequencer.

During development of the TAS assay, testing the kit on fresh produce and water samples inoculated with low levels of oocysts revealed an inability to haplotype most markers in the panel due to excessive primers dimers that were difficult to remove in sufficient quantity during purification of the sequencing libraries. To overcome this challenge, a bait-capture step was added to the workflow after the target-specific amplification (Figure 2). The barcodes and adapters for sequencing were then added to the captured amplicons in a second PCR step, creating the final libraries. Due to the nature of the sequence variation in marker CH, a bait for that amplicon is not contained in the assay. Addition of the bait-capture step greatly enhanced both the overall sequence quality and the ability to haplotype many more amplicons in samples with very low *C. cayetanensis* relative abundance.

### Genotyping substandard clinical samples

**Figure 3:**
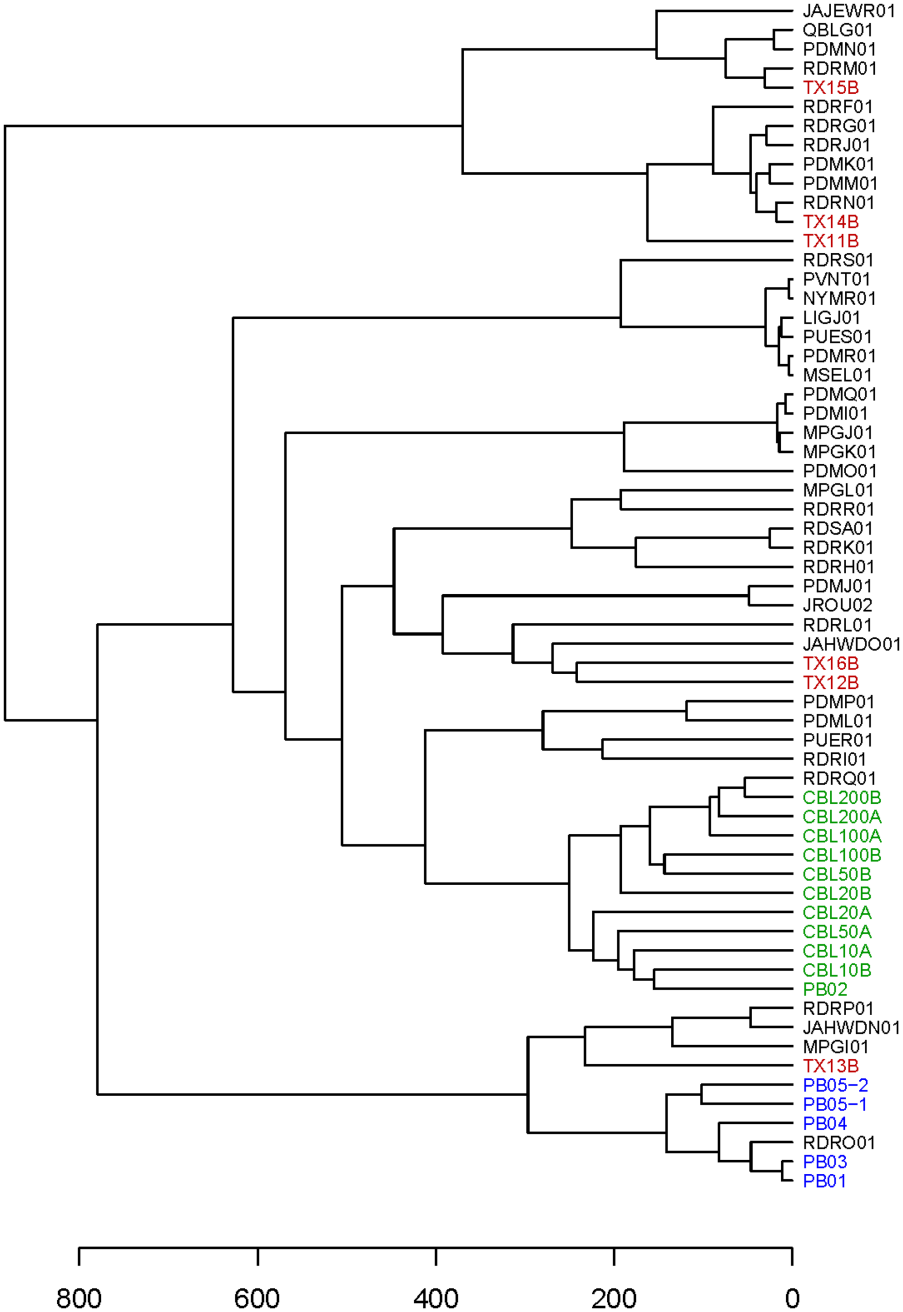
Hierarchical cluster dendrogram. The dendrogram is based on a Heuristic distance matrix generated using haplotyping results for the 52 markers in the TAS assay for the 40 *C. cayetanensis* WGS assemblies available at NCBI along with the results from all samples included in this study. The Eukaryotyping program from the CDC was used to compute the Heuristic distance matrix (Barratt et al., 2019). Accession numbers are used to label the WGS assemblies. Font colors depict: red, clinical fecal samples; green, fresh produce samples inoculated with purified Guatemalan oocysts; blue, fresh produce samples inoculated with purified Indonesian oocysts.

It was expected that all or most markers would be successfully haplotyped from clinical samples with abundant *C. cayetanensis*. Cycle threshold values for the Mit1C target in a real-time PCR detection assay had been previously determined for a selection of clinical fecal samples (Balan et al., 2023). Six of the samples had been frozen prior to DNA isolation and all but one of these samples had a measured C_T_ value above 26, the highest value for all other clinical fecal samples included in the previous detection assay work (Balan et al., 2023). The C_T_ values for the six fecal samples ranged from 25.6 to 32.4 (Table 2) and these samples were considered good candidates for evaluating the TAS assay on somewhat to very challenging clinical samples. Sequencing libraries for the six samples were pooled together in a sequencing run generating from 5.21 to 6.77 million (average 6.06 million) reads per sample. The percent reads matching to *C. cayetanensis* sequence ranged from 63 to 78% (average 70%). All sequence datasets contained less than 20 reads matching other genera in the *Eimeriidae* family. As expected, most of the markers could be haplotyped in the samples with lowest C_T_ values (Table 2), and in fact all but markers AM and CH were covered with a depth of ≥10 reads in TX14B and all but markers AK, AM, and CH in TX15B. It is not surprising that marker CH was not haplotyped considering there is no bait for that marker, thus for many samples, reads covering the other markers will be much more plentiful in the datasets. At lower *C. cayetanensis* relative abundances, fewer markers could be haplotyped. However, even for the suboptimal clinical sample TX16B, almost half of the markers, including 140 SNPs, were available for genotyping (Table 2). There were 19 markers that were haplotyped in all six samples and in most cases, when samples were missing markers, the same markers were missing (Supplementary Table S2). The Eukaryotyping program was utilized for hierarchical clustering based on the haplotyping results along with the haplotypes of the 40 WGS assemblies (Figure 3). The clinical samples do not all cluster together, suggesting different origins for the cyclosporiasis cases. For example, TX13B clusters with Indonesian isolates, whereas TX11B, TX14B, and TX15B display sequence similarity with each other, but are distant from TX13B.

**Table 2:** Targeted amplicon sequencing and real-time PCR results from frozen clinical fecal samples containing a range of *C. cayetanensis* relative abundance.

Sample	C_T_^1^	Markers haplotyped^2^	SNP sites^2^
TX11B	29.70 ± 0.04	38	241
TX12B	32.40 ± 0.79	34	230
TX13B	29.36 ± 0.07	46	347
TX14B	25.60 ± 0.08	50	387
TX15B	27.36 ± 0.17	49	379
TX16B	30.67 ± 0.26	24	140

1 Cycle threshold for real-time PCR Mit1C target, from Balan et al., 2023.

2 Number of SNP sites included in haplotyped markers. The assay includes a total of 396 SNP sites in 52 markers.

### Sensitivity determination using inoculated romaine lettuce

Having demonstrated that there was very minimal marker dropout when *C. cayetanensis* relative abundance in a sample is high as in TX14B, we sought to determine the sensitivity of the TAS assay for genotyping *C. cayetanensis* from contaminated fresh produce using inoculated romaine lettuce. The lettuce was inoculated with 10, 20, 50, 100, or 200 oocysts, and the resulting DNA was used in the TAS assay. To gain an understanding of the consistency of the assay, two technical replicates were performed for each inoculation level (Table 3). The total number of reads per sample ranged from 5.30 to 6.93 million, with an average of 5.58 million. Over 80% of the markers were haplotyped at an inoculation level of 200 oocysts, and this included over 80% of the SNP sites in the assay. For one of the replicates, all markers except AK, AV, FG, FI, and CH were haplotyped (Supplementary Table S2). At inoculation levels of 100 oocysts and below, fewer markers could be haplotyped, but never less than 24 and that still included 44% of the SNP sites in the assay. Cycle threshold values for the Mit1C real-time PCR detection assay were measured and ranged from 30.3 for inoculation of 200 oocysts to 34.6 for 10 oocysts (Table 3). The TAS assay performed well at all inoculation levels. At low inoculation levels the results reveal a lack of association between C_T_ value and number of markers haplotyped. In fact, greater coverage of the genomic variation in *C. cayetanensis* was achieved in the samples inoculated with 10 oocysts than for 20 or 50 oocysts.

**Table 3:** Targeted amplicon sequencing and real-time PCR results from fresh produce samples inoculated with purified *C. cayetanensis* oocysts.

Sample	Sample matrix	Inoculation level^1^	Oocyst type	C_T_^2^	Markers haplotyped^3^	SNP sites^3^
CBL10A	Lettuce	10	Guatemalan	34.6 ± 0.1	37	274
CBL10B	Lettuce	10	Guatemalan	34.6 ± 0.1	37	265
CBL20A	Lettuce	20	Guatemalan	31.9 ± 0.2	24	174
CBL20B	Lettuce	20	Guatemalan	31.9 ± 0.2	28	209
CBL50A	Lettuce	50	Guatemalan	32.0 ± 0.3	28	174
CBL50B	Lettuce	50	Guatemalan	32.0 ± 0.3	35	282
CBL100A	Lettuce	100	Guatemalan	30.8 ± 0.3	38	306
CBL100B	Lettuce	100	Guatemalan	30.8 ± 0.3	32	248
CBL200A	Lettuce	200	Guatemalan	30.3 ± 0.1	42	330
CBL200B	Lettuce	200	Guatemalan	30.3 ± 0.1	47	356
PB01	Salad mix	200	Indonesian	27.6 ± 0.1	50	388
PB02	Cilantro	20	Guatemalan	33.6^4^	41	297
PB03	Basil	200	Indonesian	27.5 ± 0.1	51	396
PB04	Blackberry	10	Indonesian	32.7 ± 0.3	44	322
PB05-1	Salad mix	10	Indonesian	35.8 ± 1.1	30	269
PB05-2	Salad mix	10	Indonesian	35.8 ± 1.1	40	332

1 Number of oocysts inoculated on 25 g leafy greens or 50 g berries.

2 Cycle threshold for real-time PCR Mit1C target.

3 Number of SNP sites included in haplotyped markers. The assay includes a total of 396 SNP sites in 52 markers.

4 Only one technical replicate of three had a positive detection result.

In general, there was very good consistency in the number of markers haplotyped between technical replicates performed using the same DNA sample (range of 0 to 7 markers), although there was greater variation in the difference in number of SNP positions between replicates. The greatest difference observed was seven markers representing 108 SNPs for the samples inoculated with 50 oocysts (Table 3). Sequencing depth did not explain this disparity as there were 4.97 and 5.05 million reads matching *C. cayetanensis* sequence in the datasets for CBL50A and CBL50B, respectively, the minimum difference among the inoculation levels. Comparisons between the replicates disclose a degree of variation in terms of which markers were haplotyped. For example, 37 markers were haplotyped for both replicates inoculated with 10 oocysts, however, there were 10 markers that were haplotyped in only one or the other of the samples (Supplementary Table S2). Examination of each inoculation level revealed 40, 23, 23, 16, and 32 markers haplotyped in common between the replicates for inoculation levels of 200, 100, 50, 20, and 10 oocysts, respectively. It is instructive to examine which markers were haplotyped for each of the 10 samples and compare across samples for purposes of future optimization of the marker panel design. Only seven of the markers were haplotyped in all samples, and there were an additional six markers that were haplotyped in all but one sample (Supplementary Table S2). The only marker that was not haplotyped in any of the 10 samples was CH, the mitochondrial junction region for which there is no corresponding bait.

The expectation is that naturally occurring *C. cayetanensis* contamination on fresh produce will be low, thus not all markers in the TAS assay will necessarily be amplified in acceptable quantity for haplotyping. However, it is important for linking contaminated food products to clusters of illnesses for the genotyping results to cluster genetically related samples together. We used the haplotyping results from the sensitivity assay as input into the Eukaryotyping program. The hierarchical cluster results demonstrate that the 10 samples are closely clustered (Figure 3). Furthermore, these samples inoculated with Guatemalan oocysts cluster most closely with a Guatemalan isolate, RDRQ01.

### Genotyping from a variety of fresh produce types

After investigating the performance of the TAS assay using artificially contaminated romaine lettuce, the ability to genotype *C. cayetanensis* contaminating other relevant fresh produce matrixes was assessed (Table 3). Cilantro was inoculated with the Guatemalan oocyst preparation used for the romaine lettuce, and basil, salad mix, and blackberries were inoculated with purified oocysts from a fecal sample associated with a clinical illness in Indonesia. The measured C_T_ values for the Mit1C real-time PCR target were below 30 for samples inoculated with 200 oocysts and as high as 35.8 for inoculation levels ≤20 oocysts (Table 3). The total number of reads in the sequence datasets for these samples ranged from 3.04 to 7.84 million (average 4.33 million). Including datasets for these produce samples along with the inoculated romaine lettuce samples from the sensitivity experiment, an average of 87.4% of the total reads per sample matched *C. cayetanensis* sequence. Only two samples had greater than 20 reads matching sequence to other members of the *Eimeriidae* family. There were 15,460 reads matching *Eimeria* in CBL50A and in PB04, a blackberry sample, there were 104 and 70 reads matching *Eimeria* and *Isospora*, respectively. The TAS assay performed well on the various fresh produce items evaluated (Table 3). Only one marker, CH, was missing from the haplotyping results for basil inoculated with 200 oocysts, and for salad mix, also inoculated with 200 oocysts, only markers AK and CH were not haplotyped. The TAS assay results demonstrate the ability to achieve significant coverage of loci with genomic diversity in the *C. cayetanensis* genome even at the low contamination level of 10 oocysts and in several different matrixes. The haplotyping results for these produce samples along with the romaine lettuce results above revealed six markers in common haplotyped among all samples, namely, AX, FF, CF, CG, CJ, and CK (Supplementary Table S2). Additionally, four markers, AP, FC, FE, and FN, were missing in only one sample each. Adequate sequence data was available in only 50% of the fresh produce samples for haplotyping markers AK, AO, FT, and CD, and marker CH was unable to be haplotyped in any sample.

In the workflow for the TAS assay (Figure 2), only half of the bait-capture enriched targeted amplicons are used in the indexing PCR and the second half is held as a backup. We were interested in determining whether there would be markers haplotyped in one portion of the enriched target but not the other for a low contamination sample. To this end, the second portion of the enriched target for the salad mix sample PB05-2 (Table 3) was subjected to the indexing PCR and sequenced. While 30 markers were haplotyped for PB05-1, 40 markers were haplotyped for PB05-2, and those 40 markers included all 30 markers haplotyped for PB05-1. Therefore, while there were additional markers for genotyping from PB05-2, there were no markers unique to PB05-1. PB05-2 had 1.86 million more reads matching *C. cayetanensis* sequence than PB05-1, and this increased sequencing depth is the probable explanation for the difference in the number of markers haplotyped. The haplotyping results from the variety of inoculated fresh produce samples were included as input in the hierarchical clustering analysis using the Eukaryotyping program (Figure 3). The cluster dendrogram generated reveals that sample PB02, a cilantro sample inoculated with Guatemalan oocysts, clusters with the inoculated romaine lettuce samples and the Guatemalan isolate RDRQ01. Also of significance, the fresh produce samples inoculated with Indonesian oocysts were linked genetically and clustered most closely with the WGS assembly of an Indonesian isolate, RDRO01.

## Discussion

There are currently no tools in use for genotyping *C. cayetanensis* from contaminated fresh produce or environmental samples despite the rising number of cyclosporiasis cases over the past few years in the United States (See foot note text 1). Attempts have been hampered in large part by the inability to obtain the genomic sequence diversity necessary due to the low contamination levels, which in turn result in very low relative abundances of *C. cayetanensis* in microbiomes derived from fresh produce and surface water. Both TAS and bait-capture are popular techniques for enriching loci of interest from culture independent metagenomic samples and each has particular advantages and disadvantages (Fitzpatrick et al., 2021; Singh, 2022). However, neither of these enrichment techniques independently yields the sensitivity required for including nuclear targets in a scheme for genotyping *C. cayetanensis* from fresh produce items. In this work, we combine the two enrichment techniques, performing TAS followed by bait-capture of the targeted amplicons to achieve exceptional sensitivity that allows for genotyping from many targets in the nuclear genome at very low contamination levels and with low total volumes of input DNA. Since bait-capture is performed subsequent to target-specific amplification, a rapid hybridization step of 35 min was used in this work, resulting in the ability to fully complete sequence library preparation within 1 day despite the extra steps involved. Achieving the required sensitivity with very little time sacrifice is advantageous, especially during outbreaks for which fresh produce is the suspected food vehicle. Not only does the assay provide greater resolution than previously possible for genetic linkages, but it also results in a plethora of sequence that can be used for confirmation of positive real-time PCR detection results for *C. cayetanensis* that is available even prior to the bioinformatic analysis entailed in haplotyping.

The eight marker MLST assay currently utilized by the CDC is comprised of individual conventional PCRs for each of eight markers, namely, CDS1, CDS2, CDS3, CDS4, HC378, HC360i2, MSR, and MT-junction (Nascimento et al., 2020). PCR products are then pooled for sequencing library preparation. In order for a sample to pass the criteria to be considered for successful genetic clustering, either at least five markers must have genotyping data available in the sequence datasets, or data must be available for the three markers HC378, HC360i2, and MSR, along with one of the five additional markers (Barratt et al., 2019). Success rates of 79% (Barratt et al., 2021) and 81% (Yanta et al., 2022) for meeting these minimum requirements on fecal specimens have been reported. This suggests that approximately 20% of the fecal samples received from cyclosporiasis cases present a challenge possibly due to low parasite loads. Our results for the suboptimal clinical samples demonstrate that with the TAS assay many more markers representing greater genomic diversity can be successfully haplotyped from fecal samples with low parasite loads. This improvement not only provides greater genetic resolution but will allow many samples that would otherwise be excluded from analyses involving linkages to other fecal specimens or food items to be included. Addition of bait-capture may not be necessary for genotyping clinical fecal samples with abundant *C. cayetanensis* using the TAS assay. However, performance on numerous clinical fecal samples would need to be completed to determine whether the bait-capture step could be routinely removed for most samples without negatively impacting the ability to haplotype an adequate number of markers to resolve linkages between samples. In cases where additional enrichment is not necessary, both the target-specific amplification and addition of barcodes and adapters could be accomplished in a single PCR.

Although *C. cayetanensis* has been detected on various types of fresh produce, and specific produce items have been associated with outbreaks based on epidemiological investigations (Almeria et al., 2019; Hadjilouka and Tsaltas, 2020), it has not been possible to definitively genetically link clinical specimens and food items. Additionally, it is difficult to unravel possible overlapping outbreaks due to different *C. cayetanensis* strains when relying solely on epidemiology (Barratt et al., 2022). We chose to focus our initial work on romaine lettuce as it is commonly consumed and is considered a high-risk food commodity for *C. cayetanensis* infection, particularly in the United States (Almeria and Shipley, 2021). In recent years, a variety of salads containing romaine lettuce, among other ingredients, have been implicated in outbreaks of cyclosporiasis (Almeria and Shipley, 2021), and *C. cayetanensis* contamination has also been reported in RTE and pre-packaged bulk salad mixes in Canada and Europe (Hadjilouka and Tsaltas, 2020; Almeria and Shipley, 2021). Our results using artificially contaminated romaine lettuce and salad mix demonstrate the ability to obtain sequence for many of the 52 informative markers in the TAS assay for these important matrixes even at low contamination levels. *C. cayetanensis* has also been detected on berries during surveillance activities (Temesgen et al., 2022) and has been associated with contaminated berries through epidemiology during cyclosporiasis outbreaks (Herwaldt and Ackers, 1997; Almeria et al., 2019; Hadjilouka and Tsaltas, 2020). Although the focus of the present work was leafy greens, a blackberry sample inoculated with 10 oocysts was included to verify that the TAS assay would also perform satisfactorily on contaminated berry samples. Genotyping from the blackberry sample was successful, with 322 of the 396 SNP sites included in the sequence dataset. In a report detailing the genetic clustering of fecal specimens associated with cyclosporiasis in Canada using the eight marker MLST scheme employed by the CDC, it was noted that the cluster assignment of one of the specimens changed when the sample was resequenced to cover more of the sequence included in the eight markers for genotyping (Yanta et al., 2022). This implies that, as pointed out, the number of markers haplotyped may affect genetic clustering even when the minimum requirement for the MLST scheme is met. Furthermore, as suggested in an examination of genotypes of clinical samples throughout 2020, inclusion of additional nuclear markers to the eight marker MLST scheme is expected to improve cluster stability as specimens are acquired over time (Barratt et al., 2022). We demonstrate that the TAS assay in the present work is able to reproducibly obtain sufficient *C. cayetanensis* sequence from low contamination samples to genetically link samples prepared with the same oocyst preparation. Importantly, although to some extent different numbers and combinations of markers that could be utilized for genetic clustering were obtained among the various romaine lettuce samples, the samples clustered more closely with each other and with a Guatemalan isolate than with other isolates. Similarly, the produce samples inoculated with Indonesian oocysts clustered more closely with each other and an Indonesian isolate than with the samples inoculated with Guatemalan oocysts or with other isolates despite the variation among markers haplotyped for each sample. The much greater number of informative markers in the TAS assay compared to the eight loci MLST method for computing genetic cluster dendrograms results in a higher degree of confidence in the placement of samples even when sequence data for some markers is missing.

During outbreaks or surveillance activities, genotyping would be performed on fresh produce samples that have already been determined positive for *C. cayetanensis* contamination using a real-time PCR detection method. We explored the association between the cycle threshold values obtained by real-time PCR (Balan et al., 2023) and the number of markers obtained with the TAS assay. With C_T_ values lower than 28, all but one to three markers were haplotyped. For values above 30 cycles, greater marker dropout numbers were observed, but never too many for successful genetic clustering, even for the highest C_T_ value of 35.8. Although fresh produce samples inoculated with less than 10 oocysts were not included in this study, C_T_ values measured using the same real-time PCR protocol for cilantro, raspberry, and romaine lettuce samples inoculated with five oocysts, the level of fractional detection, were reported to range from 33.8 to 37.9 (Balan et al., 2023). Together, these results suggest that the TAS assay will perform effectively for contamination amounts down to the level of detection, although experiments utilizing the TAS assay on produce samples inoculated with five oocysts would provide further confirmation.

The results of our technical replicates of the inoculated lettuce revealed overlap between markers haplotyped, but also some variation. The variation could not be explained by sequencing depth differences. In contrast, in the sequence datasets for salad mix samples PB05-1 and PB05-2 we observed no markers haplotyped in PB05-1 that were not also haplotyped in PB05-2. Rather than separate DNA aliquots, these two sequence datasets were from separately performing the indexing PCR and subsequent sequencing on the two aliquots of baited amplicons obtained while performing the TAS assay on one sample. The additional markers haplotyped for PB05-2 compared to PB05-1 could be explained by the greater sequencing depth acquired for PB05-2. It is important to emphasize that in this work, our results demonstrate that marker dropout cannot be interpreted as arising from primer and template sequence differences as is sometimes the case for TAS work, but rather as a result of the extremely low relative abundance of *C. cayetanensis* DNA in the total sample DNA. In light of these results, attaining a greater number of markers for haplotyping from a low contamination sample during an outbreak could likely be accomplished by either deeper sequencing than was used in this study, or performing TAS on two separate aliquots of DNA. In this work, 5 μL of DNA template was used in the target specific PCR, however, the protocol specifies that up to 10 μL may be used, providing another option to explore.

When applying a TAS approach to linking isolates genetically, it is important to include a suitable number of markers with high discriminatory power to resolve genetic clusters and to have confidence in the placement of isolates within the clusters. Our TAS assay greatly expands the panel of markers used for genotyping *C. cayetanensis* compared to the eight marker MLST currently in use for clinical specimens. The targeted loci were predominately chosen for informativeness using computed Shannon entropy values based on variation at known SNP sites in the *C. cayetanensis* genome. We constructed our SNP-based phylogenetic tree utilizing all core genes in the chromosomes of the available WGS assemblies, however the assemblies may contain only one allele, or a hybrid of two alleles, at a heterozygous locus. For assemblies without corresponding sequence read files, the core gene loci cannot be inspected for these potential issues. Nonetheless, with this caveat in mind, the phylogeny demonstrates the genetic clustering of isolates based on the evolution of many loci in the nuclear genome. The chromosomal core gene and TAS marker SNP-based phylogenies display a remarkable concordance. The similarity of the tree topologies suggests the number and genomic locations of the markers were well chosen. Ideally, whole genome SNP phylogeny would be used for determining the evolutionary relationships of *C. cayetanensis* isolates, however, obtaining whole genome sequences is difficult. Without complete genome information, the markers in the TAS assay will enable demonstration of the evolutionary relationship of clinical and environmental isolates. However, while the TAS assay is a major improvement on the eight loci MLST method for genotyping, the panel of markers would benefit from optimization by selecting markers to remove along with adding other informative markers. In accomplishing this, several performance aspects should be examined. The entropy change for each marker was assessed as haplotyping results were added, and ideally markers with increasing entropy values should be kept. Along with discriminatory power, haplotyping success rates of the markers were inspected. The low entropy values associated with CA, CB, CC, and CD, along with the fact that CD could be haplotyped in only 50% of the samples, suggests these four markers should be removed and replaced by other more informative markers. In agreement, in a previous report, replacement of CDS1, CDS2, and CDS4 in the eight loci MLST scheme (corresponding to CA, CB, and CD in the TAS assay) was suggested based on both low entropy values and lower sequencing success rate (Yanta et al., 2022). Another consideration is that mitochondrial DNA evolves at a different rate than chromosomal DNA and it may be advantageous to capture the diversity in both in a genotyping marker panel for linking isolates during an outbreak. Currently, CG, CK, and CH target loci in the mitochondrial genome, although there is no bait for CH. It is worth assessing the value of CH to determine whether to attempt to design a bait for it or to remove it from the panel. While the current design is useful, taking all of these marker performance characteristics into consideration, along with evaluation of several new markers to add to the panel, we are working to revise the design.

The present study demonstrates that the TAS assay is very successful for genotyping difficult fecal samples and fresh produce samples inoculated at low oocyst levels. Ideally, the TAS assay would also be applied to environmental samples (water, soil, and sediment) for genetic linkage to cases of cyclosporiasis or fresh produce samples. In particular, water may act as a possible mode of transmission for contamination of fresh produce by *C. cayetanensis* since the parasite has been detected in water sources (Ortega and Sanchez, 2010; Almeria et al., 2019; Chacin-Bonilla and Santin, 2023). An added complication with using TAS for genotyping *C. cayetanensis* contaminating water is that *Eimeria* and *Isospora* species, parasites closely related genomically to *C. cayetanensis*, are found in many agricultural surface water sources, resulting in possible co-amplification of these genera, thus reducing sequencing depth for *C. cayetanensis* if TAS primers are not explicitly designed to avoid this issue. In this work, primers that matched *Eimeria* sequence too closely were excluded, and the primer panel was evaluated during development utilizing inoculated surface water samples specifically for this reason. We encountered very few reads matching other *Eimeriidae* in the datasets generated from fresh produce samples in this work and although the abundance of related genera is much higher in some water sources than on fresh produce, we anticipate that the TAS assay will prove useful for agricultural surface water surveillance activities.

## Conclusion

Development of a genotyping tool for *C. cayetanensis* with the sensitivity to perform at the very low contamination levels encountered on fresh produce is a priority for reducing cyclosporiasis illnesses and determining possible prevention strategies. The method we have developed and evaluated in this work, consisting of TAS followed by bait-capture, encompasses both the genomic diversity and exceptional sensitivity to genetically link clinical fecal specimens with contaminated fresh produce. Utilizing romaine lettuce, salad mix, basil, cilantro, and blackberries inoculated with *C. cayetanensis* oocysts, we demonstrated the ability to genotype and successfully genetically cluster down to as low as 10 oocysts per sample. Along with food samples, the TAS assay will prove useful for genotyping otherwise challenging clinical samples with low parasite loads, allowing inclusion of additional samples for source tracking analysis, a potentially difficult task when outbreaks involving separate strains overlap temporally. Our assessment of the performance of the 52 markers included in the present TAS panel design will aid in determining markers to add or remove to increase discriminatory power as the panel is further optimized. Overall, this work establishes for the first time the ability to genotype *C. cayetanensis* from fresh produce at low levels and with the genomic resolution required to complement epidemiological investigations. Furthermore, as a molecular surveillance tool, the method may be used to investigate the dispersion of *C. cayetanensis* in the agricultural environment.

## Data availability statement

The data presented in the study are deposited in the NCBI Sequence Read Archive repository, accession number PRJNA952552.

## Author contributions

SL, MM, DL, MT, and SM: conception and design of the study. CW, ZM, and BG: development of the TAS assay. ZM and SL: testing TAS assay during development. SA: real-time PCR and preparation of produce samples. SL: writing first draft of the manuscript and TAS experiments. MM and CW: bioinformatic analysis. MM: haplotyping and genetic clustering. All authors reviewed and edited the manuscript and approved the submitted version.

## Funding

This work was supported by the U.S. Food and Drug Administration and Chapter Diagnostics.

## Conflict of interest

CW, BG, and ZM were employed by Chapter Diagnostics.

The remaining authors declare that the research was conducted in the absence of any commercial or financial relationships that could be construed as a potential conflict of interest.

## Publisher’s note

All claims expressed in this article are solely those of the authors and do not necessarily represent those of their affiliated organizations, or those of the publisher, the editors and the reviewers. Any product that may be evaluated in this article, or claim that may be made by its manufacturer, is not guaranteed or endorsed by the publisher.

## Author disclaimer

The findings and conclusions in this article are those of the authors and do not necessarily represent those of the U.S. Food and Drug Administration.

## Footnotes

^1^ www.cdc.gov/parasites/cyclosporiasis/outbreaks/index.html

^2^ http://www.bioinformatics.babraham.ac.uk/projects/fastqc/

^3^ https://CRAN.R-project.org/package=cluster
