# Supplementary material for: Development of a targeted amplicon sequencing method for genotyping Cyclospora cayetanensis from fresh produce and clinical samples with enhanced genomic resolution and sensitivity
Source: Front Microbiol. 2023 Jun 16;14:1212863. doi: 10.3389/fmicb.2023.1212863 (PMC10311907; doi:10.3389/fmicb.2023.1212863)
Supplement: Supplementary file 1 [file Image_1.pdf]

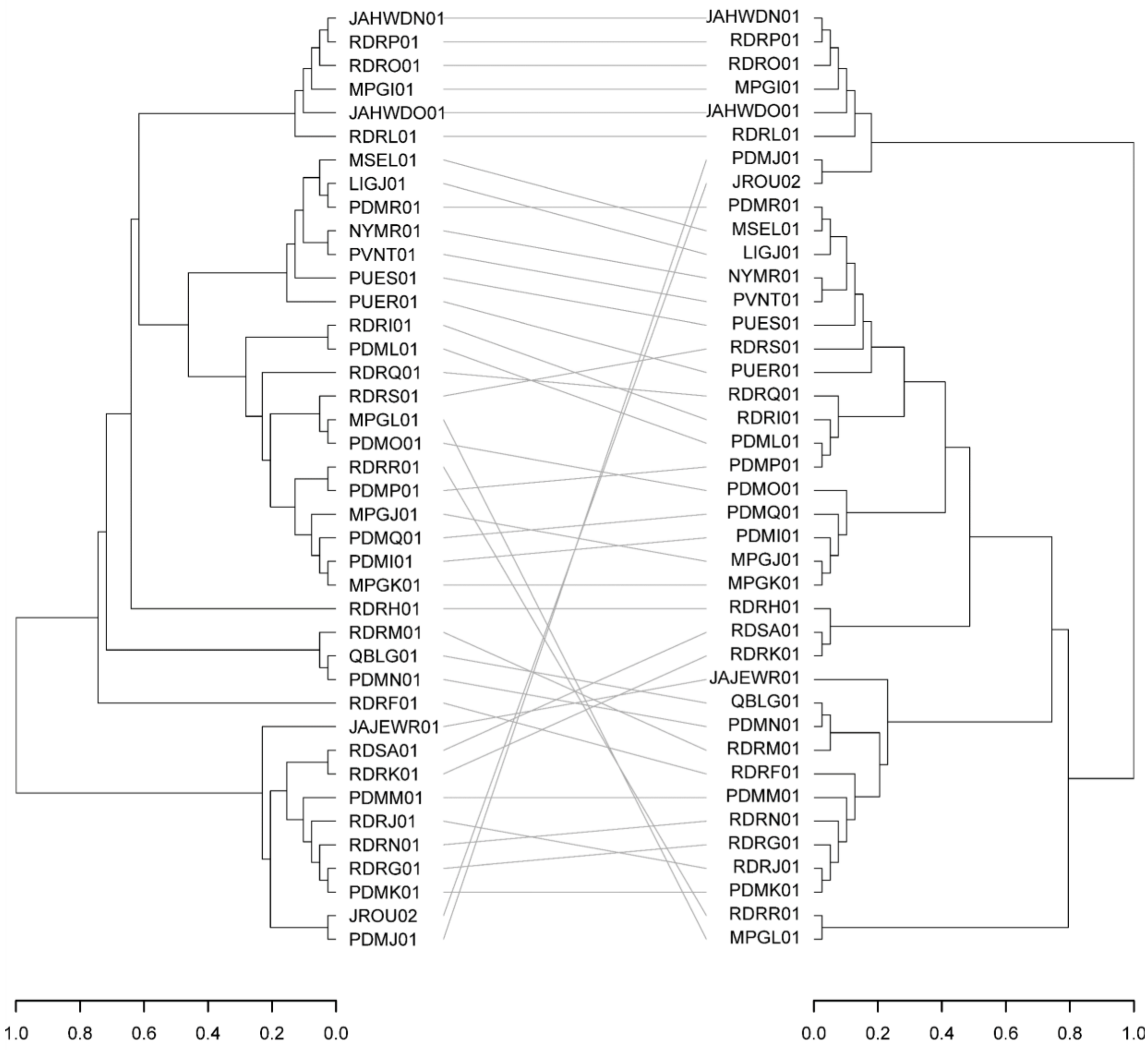

Figure S1. Visual comparison of the SNP-based phylogenetic trees generated for Figure 1. The phylogeny created using all core chromosomal genes (Figure 1A) is on the left and the phylogeny created based on the TAS assay (Figure 1B) is on the right. Lines connect the same isolate in the two trees. The comparison was generated using the R package dendextend.
