# Supplementary material for: Development of a targeted amplicon sequencing method for genotyping Cyclospora cayetanensis from fresh produce and clinical samples with enhanced genomic resolution and sensitivity
Source: Front Microbiol. 2023 Jun 16;14:1212863. doi: 10.3389/fmicb.2023.1212863 (PMC10311907; doi:10.3389/fmicb.2023.1212863)
Supplement: Supplementary file 2 [file Table_1.pdf]

Table S1. Markers in the targeted amplicon sequencing assay that overlap genome location with the eight loci MLST method for genotyping and number of SNP sites included.

MLST marker	TAS assay marker(s)	SNPs in MLST	SNPs in TAS assay	SNPs in common
CDS1	CA	7	12	7
CDS2	CB	1	2	1
CDS3	CC	1	2	1
CDS4	CD	3	4	3
HC378	CF, CJ	17	18	17
HC360i2	CE, CI	24	29	20
MSR	CG, CK	5	5	5
MT-junction	CH	NA	NA	NA

NA = not applicable
